# Veteran welfare past and present: a sociological analysis of the Civil War Petitions, 1642–1718

**DOI:** 10.3389/fsoc.2025.1495269

**Published:** 2025-03-13

**Authors:** Andrew Hopper, Ismini Pells, Martin Bricknell

**Affiliations:** ^1^Department for Continuing Education, Rewley House, University of Oxford, Oxford, United Kingdom; ^2^Faculty of Social Science and Public Policy, Bush House, North East Wing, King's College London, London, United Kingdom

**Keywords:** pensions, military, welfare, Civil War, soldiers, war widows, veterans

## Abstract

This article examines the idea of a social contract between the armed forces and the state through a cross period comparison between the United Kingdom in recent years and England and Wales during the mid-seventeenth century. In doing so, it provides an analysis, grounded in sociological theory, of an early military pension scheme evidenced by thousands of surviving petitions for military welfare made by the maimed soldiers, war widows and orphans of the British Civil Wars (1639–1652). Through the findings of the Civil War Petitions project (https://www.civilwarpetitions.ac.uk), this article provides an overview of how seventeenth-century soldiers and war widows operated from the perspective of successive government regimes, administrators, and recipients. The project demonstrates that the extent of military welfare for maimed soldiers, war widows and orphans was greater than previously supposed. In three thematic sections, this article discusses the conditions, process, and purpose of granting military pensions and monetary support. This national provision of military welfare was an important early milestone in securing popular participation in the formation of the modern fiscal-military state. Throughout, these analyses are compared with the experience of the United Kingdom's Armed Forces today, in order to assess similarities and differences with the seventeenth century, linking the experience of veteran welfare in the past through to the present. In the seventeenth century, those poor maimed soldiers and war widows who were denied pensions often found themselves dependent on parish poor relief and the charity of their neighbors. In contrast, today many veterans benefit from well-organized UK military charities, which help to compensate for the shortcomings of state welfare. The activities of these organizations continue to support a form of social contract between the armed forces and the civilian population, where the state is not always the primary link or sole provider of support. New theories of the social contract should take this plurality into account.

## Introduction

The focus of the Civil War Petitions Project on the human costs of a war undertaken nearly 4 centuries ago has enormous contemporary relevance in an increasingly uncertain world. This is because western policymakers, especially in the United States, tend to repeatedly underestimate the long-term human and financial costs of war, especially those relating to post-war veteran welfare (Fuzal, 2024, pp. 3–4, 16, 154). Many similar questions continue to surround veterans' benefits today as in the seventeenth century: who deserves what, when, how much, why, and from whom? Fuzal reminds us that care for veterans and their families “is an inevitable cost of war, and rightly so. The decision to go to war should, therefore, be conditioned by that inevitability” (Fuzal, 2024, p. 44). The legacy of the United Kingdom's combat operations in Iraq and Afghanistan, which resulted in the deaths of 179 and 457 British Forces personnel respectively, as well as the physical and psychological wounding of tens of thousands more servicemen and servicewomen, continues to be felt today (AFCS, 2024). Although public interest in veteran welfare declines sharply in peacetime, the challenges facing the wounded and bereaved remain problems that depend upon the involvement of charities and the wider civilian community to overcome. A sense of personal stigma often prevents veterans from accessing welfare services, particularly in relation to the mental health consequences of war (HCDC, 2011).

The Armed Forces Covenant, established in its present form in 2011, is a commitment from the United Kingdom government, businesses, and other organizations to support and treat fairly those who have served in the Armed Forces and their families (AFC, 2011). The origins of this commitment are traced to the reciprocal agreement between the state and those who fight in its service that was first recognized in England in the pension scheme for maimed soldiers that was established in 1593. However, it was during the Civil Wars that this scheme was overhauled to operate on a truly effective national scale, with the Long Parliament extending pension entitlement to war widows and the families of those slain in its service. The birth-pangs of today's Armed Forces Covenant are clear in the statement from the parliamentarian army chaplain, Robert Ram: “They that have received any hurt or loss by the wars ought to be liberally provided for, and comfortably maintained all their days, by them that set them forth” (Ram, 1644, p. 28).

### About the project

Recent estimates suggest that up to 3% of the population of England and Wales died as a direct result of the Civil Wars (Gentles, 2007, pp. 436–437). This was a greater proportional loss of people than Britain suffered during both World Wars combined. With the kind support of a standard grant from the United Kingdom's Arts and Humanities Research Council, in 2017, the “Conflict, welfare, and memory” project set out to uncover the human stories of suffering, loss, survival, and hope that lie behind this stark statistic of the costs of war. The core project team comprised Andrew Hopper as principal investigator, Ismini Pells as project manager, along with co-investigators David J. Appleby, Lloyd Bowen, and Mark Stoyle, ably supported by several research assistants and an IT team from the University of Nottingham. Over the last 6 years the project has digitized on a free-access, searchable website the original, hand-written petitions which thousands of wounded ex-soldiers, war widows, and other dependents submitted in order to gain pensions for which they were permitted to apply by parliamentary legislation. This article provides an introduction to the project and discusses its resonance for veteran welfare today.

These petitions, drawn-up between 1642 and 1718, provide graphic testimonies of what it felt like for ordinary people to live with horrific wounds, trauma, suffering, and loss. The petitions are sometimes supported by hand-written certificates composed and signed by medical practitioners, army officers, or the neighbors of the claimants in order to support the deserving nature of their cases. Records of how much each claimant received, where and when, have also been entered from surviving records of the courts which deliberated on pension claims and the accounts of each county's treasurers who administered the payments. These records are scattered across England and Wales in the collections of our wonderful county record offices, without whose support and collaboration, this project's research would have been impossible. Our objective was to unite these records in one searchable, digital collection that could be used by university academics and students, teachers and schoolchildren, museum professionals, civil war enthusiasts, researchers and genealogists alike. We hope this will widen discussion of the social, economic, and cultural consequences of the Civil Wars and the impacts of these conflicts on people's everyday lives.

Our project investigated the strategies used by and on behalf of maimed soldiers and war widows when they made their cases to obtain charitable relief. We showed how petitioners represented military service, how they fashioned themselves as “deserving cases,” how they played upon the expectations of the authorities, and in some cases how petitions reflected feelings of entitlement. The petitions have helped us to learn more about the many ways in which participants and victims endeavored to sustain themselves through these difficult times. The perspectives of the poor in the memory of national events are often lost, but our documents have offered a unique opportunity to hear those voices speak, albeit through the mediation of a legal process. This is as close as the early modern historian might get to a kind of “Mass Observation Archive” on the events of the 1640s and 1650s in England and Wales.

The pension scheme was organized on a county basis across England and Wales. It was administered by the courts of quarter sessions, which were the principal engine of local county government, presided over by justices of the peace drawn from among the landowning social elite. Military welfare in Scotland was organized separately by the Kirk, the Presbyterian Church of Scotland, who administered the collection and distribution (Langley, 2017). There is less surviving evidence for arrangements in Ireland, although we know that Irish soldiers in the service of the Spanish Army of Flanders were able to claim pensions at this time. Owing to the recurrent warfare across Europe during the Thirty Years' War (1618–1648), many other European states were also beginning to develop systems of military welfare. In Sweden, Gustavus Adolphus established a “Donation Book” for voluntary contributions from 1622, while a Veterans' Home Fund (*Krigsmanshuskassan*) was established to provide maimed soldiers with barrels of grain and basic relief. From 1640, maimed soldiers and officers could apply for a place at the Valdstena Hospital, a former monastic building that was set aside for wounded veterans and their families (Petersson, 2014, pp. 189–192). In Scotland in 1634, Colonel Robert Monro established a fund of voluntary contributions from officers to pay for a projected new military hospital in Edinburgh. Subsequently, by the 1640s, care for sick soldiers was the first item among Alexander Leslie's Articles of War for the Army of the Covenant (Murdoch, 2016, p. 61). The factors influencing the ways in which military welfare emerged across Europe at this time and the types of provision which resulted are subjects currently under investigation (Pells and Rommelse, forthcoming).

### Sociological theory about state provision of welfare to the military

Sociological theory concerning the state's provision of welfare to its armed forces is very much about the regime demonstrating its legitimate right to rule. This is especially critical in the aftermath of prolonged civil war. From the 1640s to the 1660s the Long Parliament, Protectorate, and Restoration regimes all felt unstable and vulnerable to domestic enemies. A system of military pensions to reward their supporters not only facilitated the recruitment and maintenance of national standing armies from 1645, it also drew the ratepayers who financed it into participation in a process of state formation as described by Charles Tilly (Lachmann, 2020, pp. 458–459). For Tilly, despite significant variation within Europe, nation states developed largely through the roles of coercion and capital in mobilizing their populations and taxpayers for war (Tilly, 1990). This effort developed institutions that played a leading role in state formation, as seen in Tilly's famous observation: “War made the state, and the state made war.” More recently, scholars have questioned whether the British state emerged in response to international warfare, postulating that party debate and political choices were more important in shaping this process (Pincus and Robinson, 2016, pp. 229, 261).

The fiscal-military state that emerged in early modern England, as argued famously by John Brewer, did so as England became more efficient at extracting money from its people to finance prolonged warfare (Brewer, 1989). Although often associated with the wars against Louis XIV in the 1690s, a case might be made to relocate early aspects of the emergence of an English fiscal-military state to the 1640s. At enormous human and financial cost, forces loyal to the English Parliament completed a conquest and occupation of Ireland and Scotland during the 1650s. On 30 September 1651 Parliament passed an act to provide for those of its soldiers who were wounded in conducting those campaigns, as well for the widows and families of those slain.

The pension scheme was underpinned by national taxation in England and Wales. This was formalized by parliamentary ordinance from 28 May 1647, with every parish being set a weekly contribution. It raised and dispersed hundreds of thousands of pounds in relief over the next 3 decades. In order to function, it required the collaboration of national institutions such as the Parliament and its panoply of committees, as well as local institutions such as each county's quarter sessions' courts. It also required the participation of local office-holders such as justices of the peace, county treasurers, high constables, parish constables, army officers and medical practitioners. The return of the royalist gentry into national political life during and after 1660 was about a reassertion of their control over the state's institutions and offices of local government. The pension scheme was retained under a new act of June 1662, but was in theory restricted to those who had “always been loyal” to Charles I or II. Part of this included an intention to dominate the public memory of the wars, by determining who precisely the pension scheme would reward, when, where, and by how much (Bowen and Stoyle, 2021).

The idea that conscription transformed subjects into citizen-soldiers who saw themselves as part of a nation is most usually associated with the American and French Revolutions. Yet mass impressment into Parliament's New Model Army, and its growing identification as a force for imposing Englishness (Stoyle, 2005), might be argued to have anticipated these developments by over a century. Likewise, when Union veterans and their families were rewarded with pensions in the aftermath of the American Civil War, in the wake of them having “demanded moral recognition of their sacrifices,” few of them might have imagined that their English and Welsh forbears had acted similarly 2 centuries earlier. Therefore, Tilly's process of rulers offering subjects social benefits to bind them to the state as citizens was occurring earlier—if on a smaller scale—than is sometimes allowed (Lachmann, 2020, pp. 474–475). The idea of reciprocal obligations began to be established; those who fought for the state, either in the shape of the Long Parliament, the Commonwealth or the Stuart monarchy, now might expect something from those authorities in return (Kujala and Danielsbacka, 2018, p. 98). When Abraham Lincoln pledged a steadfast commitment “to care for him who shall have borne the battle” (Fuzal, 2024, p. 3), the President echoed the religious concerns for naval widows expressed by Richard Deane, General at Sea, in 1653: “Victory is purchased with the blood of those who are precious in the eyes of the Lord” (Hudson, 1994, p. 148).

## Methods

The research method employed by the Civil War Petitions project's team was one of archival historical research, followed up by web-publication accompanied by quantitative and qualitative analysis. The project team's investigators visited The National Archives at Kew for State Paper records (TNA, SP 16, SP 18, SP 28 and SP 29), and nearly every county record office in England and Wales for quarter sessions records (for example WYHC). During these visits, they identified the pertinent documents and compiled lists of them for digitization. The archival staff performed the required scanning and photography. The documents were then transcribed individually by members of the project team and the transcriptions were checked by the project manager. Then the photographs and transcriptions were uploaded to the project's Sharepoint from where they were published on a county-by-county basis onto the project's website designed by the Multimedia Online Data Service at the University of Nottingham: www.civilwarpetitions.ac.uk.

The website can be searched by places, payments, military engagements (events), names of persons, types of wounds, ailments, and injury locations. County summary pages provide tables of the mean average of pensions or one-off gratuities that were awarded to different types of claimants. This quantitative analysis was calculated automatically by the programming underpinning the website's data. County summaries also provide maps of the geographical distribution of claimants and explanatory commentary. Users can also search all content by keywords, while on search results pages, they can narrow and refine their results by using filters. The website uses “Lucene Search.” An “Advanced Search” allows them to develop complex searches, while “Fuzzy Search” and “Wild Card” functions are also available (Using Search on Civil War Petitions).

Justices of the peace and onlookers in public courtroom environments evaluated the veracity of Civil-War stories of suffering and loss every decade from the 1640s until only a handful of veterans remained in the 1710s. This continual process of talking about wartime experiences by petitioning and reapplying for pensions reinvigorated wartime memories and circulated them to new audiences, especially among younger generations. For the purposes of this article, the surviving data allows qualitative analysis of firstly the conditions that claimants had to meet in order to make a successful claim, secondly the processes through which they applied for and were awarded military pensions, and thirdly, the motives of the state in granting or denying them aid.

## Materials

The doctoral research of Geoffrey L. Hudson pioneered the study of the English county pension scheme, first established in 1593 (Hudson, 1995). Hudson demonstrated that the costs of military welfare increased tax burdens on civilians significantly from the 1640s to the 1670s (Hudson, 2000). He was also the first to recognize that the Long Parliament's landmark provision of entitlement to pensions for war widows in 1642 inserted women into a special category previously populated only by men. Now, for the first time, war widows merited relief, not solely for pious or charitable reasons, but because of recognition for their contribution to the state and participation in the political nation. Parliament's purpose for this was to win popular acceptance of their legitimacy as a regime (Hudson, 1994, p. 147). Two research articles followed Hudson's work in examining the county pension scheme on a more local level, comprising county studies of Essex (Appleby, 2001) and Devon (Stoyle, 2003). Furthermore, Eric Gruber von Arni, and more recently Ismini Pells challenged previous condescending assumptions about the quality of seventeenth-century military medical care, defending the proficiency of contemporary practitioners and the efficiency of Parliament's new military hospitals (Gruber von Arni, 2001; Pells, 2019). Two more doctoral theses helped pave the way for the “Welfare, conflict, and memory project,” each examining the operation of the pension scheme in southeast England (Worthen, 2017) and the midlands (Beale, 2018). A third doctoral thesis compared narratives of harm and loss in English and Welsh petitions with witness testimonies among Ireland's 1641 Depositions (Muckute, 2023). Research on military welfare in the twentieth century has revealed how, after a gap of over 2 centuries, war widows of non-commissioned officers and soldiers were once again allowed to claim state pensions as a result of the South African War in 1901 (Riedi, 2018).

## Results

Currently the Civil War Petitions website includes 2,229 petitions, 991 certificates, records of 26,850 individual payments to maimed soldiers, war widows, and other dependents of civil-war soldiers, as well as the names of 20,899 historical individuals. These people include ~10,200 soldiers, 1,800 war widows, and 500 orphans and bereaved dependents. Once dissipated across 40 English and Welsh county record offices, the records of Civil-War related military welfare are now all united in one digital location. The data can now be analyzed to calculate the mean average pensions awarded in each county to maimed soldiers, war widows and orphans by the parliamentarian authorities up until 1660, and by the royalist authorities thereafter until our last known surviving payment in 1718. We need to exercise caution with quantitative analysis of these statistics because the records that survive are only partial. Some counties, such as Bedfordshire do not have any surviving documentation at all, and others such as Hertfordshire have only partial records referring to particular years or subdivisions within the county. The evidence is disappointingly small for East Anglian counties, which recruited large numbers of soldiers for Parliament's war effort. Deliberate destruction of these records after the Restoration remains a possibility; parliamentarian officials did not wish their involvement with the regime to be evident, or to supply the returning royalist justices with lists of soldiers who had been loyal to the Republic. The largest collections of petitions and certificates survive for Cheshire (546), Lancashire (422), Devon and Exeter (359), and Denbighshire (287). More generally, we are often able to observe how the amount awarded reflected not just the severity of the claimant's disabling wounds or level of need, but also their social status and the availability of funds.

Among what records do survive, we can see which counties funded the largest numbers of claimants, and how generously. For example, the quarter sessions' order books for the North and West Ridings of Yorkshire are particularly detailed and extensive. They show that a total of 1,734 claimants were granted pensions or one-off gratuities between 1645 and 1710. Mean averages for each category of claimant were calculated by dividing the number of individuals relieved by the total cumulative sum awarded. Yorkshire's parliamentarian soldiers received mean annual pensions of £2, 4 shillings and 8 pence in the North Riding, compared to only £1, 17 shillings and 9 pence in the more populous West Riding. These compared to a mean pension of £2, 2 shillings and 5 pence for North Riding war widows of parliamentarians and £1, 4 shillings and 5 pence for war widows of West Riding parliamentarians. Royalist pensions were slightly lower as they applied in greater numbers—and for more years—after 1660. North Riding royalist soldiers received a mean pension of £1, 4 shillings and 10 pence, and West Riding royalist soldiers a mean pension closer to their parliamentarian counterparts of £1, 17 shillings and 3 pence. The mean pensions of royalist war widows in the North Riding were only two thirds of the value of their parliamentarian predecessors, at £1 and 8 shillings. In several countries post-Restoration justices seem to have been reticent about awarding annual pensions to widows of rank-and-file royalist soldiers, perhaps believing that it was time for women to return to their pre-war status, lose their entitlement to regular pensions and instead revert to parish welfare as they had done before 1642 (Hopper, 2025).

Turning to broader qualitative analysis, the evidence suggests that military medical practitioners were successful at keeping a great many very seriously wounded soldiers alive. Many of them survived and lived for many decades afterwards, carrying terrible wounds that modern medical military historians might sometimes wrongly suspect to have been fatal without the advantage of antibiotics and anesthetics (Appleby and Hopper, 2018). In 1668, Edward Bagshaw of Conisbrough petitioned that at Marston Moor he did “receive many wounds & cutts in the head in somuch that your peticioner had nine bones taken out of his skull, And all that nourished your peticioner for 3 weekes he received in att a hole in the side of his head & was shott, into the side of his body att the same tyme” (Bagshaw, 1668). Despite these horrendous wounds, Bagshaw was alive to petition 24 years later. In 1644, John Barrett, a corporal in the Gloucester garrison, petitioned that he had “receved seven wounds in the head five of them therow the scull one cut in the backe (to the bones) with a pole ax, his elbow cut off bones and all: his hand slitt downe betwine the fingers as Mr. Caradine the Cyerrugion afermeth who hath almost Cured them all (and very carefully and willingly he hath taken the pains to do it) how to satisfie him we know not, he was never the man that asked us a farthing” (Barrett, 1644). These cases, and many others that are similar, challenge modern assumptions that seventeenth-century medical practitioners were dangerous quacks or charlatans (McCallum, 2008, pp. 291–294; Gabriel, 2013, pp. 65–85). Researchers interested in wounds and their treatment can refine their searches into the parts of the body where wounds were sustained, and the types of injury that were inflicted, for example by gunshot or blade.

The petitions from war widows and orphans provide further reminders of the suffering and human costs of war. When long absent soldier-husbands did at last return, they were often too wounded to work and required care at home (Gruber von Arni and Hopper, 2016). Wives and mothers were confronted by returning male relatives traumatized by war. One Dorset widow, Margaret Buckler, lost her husband in Parliament's service, and was left to provide for her son who returned from soldiering “and is now lunatique.” She petitioned the county committee that she was unable to care for him and they ordered her a gratuity of £5 (Buckler and payment, 1646). Fears of starvation and homelessness abound in the petitioning narratives of many poor war widows. Failure to pay rent, sleeping rough, denial of charity, and the image of doors closed against them also loom large.

The Elizabethan Poor Law of 1601 granted responsibility to grandparents to keep orphaned children if the surviving parent was unable and the grandparents were “of sufficient ability.” Uncles and aunts were morally expected to help in such cases, but were not legally required to do so (Houlbrooke, 2013, pp. 218–219). Orphans with no relatives willing to care for them were dependent upon whatever poor relief their parish could provide for them. When William Ravenscroft was killed fighting for Parliament at Warrington in 1643, his widow died in childbirth soon after and the unnamed infant was left in the care of a kinswoman, Margaret Ravenscroft. Margaret petitioned the Cheshire justices in 1651 that she was now too poor to continue looking after the child, remarking it would be a “great pittie” for her, “after so long succor to be forced to expose it to famishing & begging.” Margaret begged the justices “in the name of this poore friendless fatherless & motherless orphant and for gods Cause that your honnor will be pleased to sett downe such order and Course for the future as some provision of Releife may bee made.” The justices assigned the child an annual pension of 40 shillings and ruled that the child should be returned to the parish of their birth and Margaret discharged from relieving them (Ravenscroft, 1651).

Where evidence of the claimants' home localities survives, either in a petition, certificate, or order book, we have mapped their locations by county. This resulted in the conclusion that in many regions both sides were recruiting from the very same communities. This meant that when soldiers returned home after the wars, they had to live side by side with their former enemies. This has led us to rethink the depth and persistence of post-conflict culture in England and Wales. Researchers can also search for the wounded and widowed by particular battles and military engagements. Analysis of the results for the Battle of Naseby in Northamptonshire in 1645 reveals a very clear east vs. west split between the armies. Fifteen of the 38 royalist claimants who mentioned being wounded at Naseby were Welsh with a further 12 coming from western English counties bordering Wales such as Cheshire and Herefordshire. This compares to the six parliamentarian claimants, largely drawn from Essex and Hertfordshire.

## Discussion

War can have devastating consequences for the physical, mental, and social health of the combatants, their family members and, indeed, wider civilians. While some veterans survive war relatively unscathed, it is inevitable that those most exposed to combat will be at risk of significant physical trauma, conflict-related mental ill-health, and difficulty in adjusting back to their civilian social networks—including work and family. This reality is self-evident, but it can be difficult to emotionally connect to the meaning and the impact of war without hearing from those personally affected.

While war creates significant technical challenges for the medical services, the most demanding difficulties for patients and their families come later as veterans go through rehabilitation, recovery, and re-integration back into civilian life. This is the real test for the social contract between the fighter and the state on whose behalf they fight. Appropriate long-term care is frequently difficult to identify and always costly. Governments may be reluctant to commit adequate resources to meet these needs and may lack the support to do so from wider society, especially if the latter does not identify with the conflict fought in their name. Failure to provide for those who have sacrificed life and limb in conflict can create feelings of injustice from the wounded and bereaved, as well as distrust toward governments and society. The social contract thus risks fragmenting. During the early twenty-first century, the United Kingdom saw political and public evolution in the accountability between the state, the Armed Forces and veterans in the provision of care and support to those most harmed by war. This is most evident through the publication of the Armed Forces Covenant in May 2011. This set out the principle that those who serve in the Armed Forces, whether regular or reserve, and those who have served in the past and their families, should face no disadvantage compared to other citizens in the provision of public or commercial services. Special consideration is appropriate for those cases who have given the most—especially those who have been injured or bereaved.

So how does a state compensate for the consequence of personal loss from war? Of course, the first thing might be to compensate for loss of life. It is clear that spouses and children of soldiers who have been killed in military service lose access to the financial sustenance from their service member and therefore might receive a war pension. One example is the order for Alice Palmer of Warkton from 14 July 1664, which recounted how her husband, John Palmer, a royalist soldier had died, leaving his wife and seven children with insufficient livelihood to survive. Through Alice's petition, she was awarded £2 yearly (Palmer, 1664). Taking us to modern times, state provision for war widows of non-commissioned officers and soldiers was only introduced in 1901, toward the end of the South African War. Both the mechanism for award and the level of award has varied according to political priority and balance with the wider statutory pensions and civilian provision. The most recent scheme, the Armed Forces Compensation Scheme, was introduced in 2005 and combined the War Pension Scheme and the Armed Forces attributable benefit scheme, and also addressed some anomalies that were exposed through the application of the previous schemes to the bereaved and injured from operations in Iraq and Afghanistan.

The second form of state compensation might be to compensate for loss of health. This is more complicated than loss of life because the assessment also requires a measure of disability alongside a measure of attribution. Such measures might include the petitioner's description of their experience, observation of physical signs and reports from medical experts. One example from the Civil Wars are the certificates for William Gray of Braintree, one of which is a certificate for his military service and the other is a certification of his injuries signed by a surgeon (Gray, 1657). Taking us into the modern day, the AFCS (2005) compensates for war injuries, including now enabling claims for personnel who are still serving. Payment can be by lump sum or, for more severe injuries, by a guaranteed income payment, and the information required includes the service person's details so as to demonstrate their military service; their medical history; details of their current doctor, and any other supporting documents. The actual award is based on an assessment done by a panel and graded according to the severity of the injury by body part and functional outcome.

### The conditions for a successful claim

Applicants for military welfare from the 1640s had to meet several expectations and conditions for their claims to be successful. First of all, claimants had to prove that their injury or bereavement had occurred in military service. Secondly, they had to demonstrate how this prevented or disabled them from working. Thirdly, there were political considerations. Claimants had to demonstrate they had served the government in place at the time. Until 1660 this meant the successive parliamentarian and Interregnum regimes, and after 1660 the restored monarchy of Charles II. The local justices had ways of finding out if soldiers misrepresented their service. There were plenty of informers prepared to “discover” such claimants, especially if they could then claim the forfeited pension themselves. In 1648 Matthew Thackwray informed against two of his neighbors—George Thackwray and Henry Leigh, who were claiming pensions as parliamentarians. He contested that they had actually been in the King's service, and consequently their pensions were removed, enabling him to claim a pension in their place (Thackwray, 1648). From 1662, royalist claimants had to prove that they had always been loyal to the crown; therefore side-changers—of whom there were thousands—were ruled out of receiving any support. Pensions might also be forfeited for failing to conform to the justices' expectations. For example, Lawrence Key lost his pension in 1683 because, in his own words, he had been “seduced to turn Quaker.” He confessed that his wife was responsible. After he had returned to the Church of England and promised never again “to be led away by a silly woman,” his pension was restored (Key, 1683). Many communities could be very supportive of claimants, endorsing their petitions and supporting their claims with signatures and certificates. Ratepayers often hoped to shift the burden of maintaining a petitioner away from poor relief in their own parish and onto a pension to which the whole county would contribute. However, if neighbors felt that a claimant was unworthy, they could prove obstructive. Nathaniel Maund was petitioned against by his local community who judged that he was capable of working but just chose instead to beg. They accused him of drunkenness, swearing, and abuse toward his neighbors, and were successful in removing his pension (Maund, 1680).

Petitioners could be quite sophisticated in presenting themselves as deserving cases to the justices. The scribes who drafted their petitions knew the relevant legislation well, including what keywords and phrases to cite. This included a deferential address at the beginning, and then a promise to pray for the long life, health, and prosperity of the justices in the closing appeal. Sometimes petitioners and their scribes tailored their narratives to suit specific individuals in authority. By claiming to be a “true member of ye High Church of England,” Henry Norton appealed to the religious preferences of Northumberland's Tory justices in 1710 (Norton, 1710). Similarly in petitioning Lord Protector Oliver Cromwell in 1655, Jane Meldrum, the widow of a Scots parliamentarian colonel killed in 1644 knew how to draw on Cromwell's godly millenarianism. She requested, successfully, “that your highness wilbee gratiously pleased to Number Her amongst your distressed widdowes whom God hath drawne forth of your pious heart mercifully to relieve. And Christ will put it to your Accompt on the Great day” (Meldrum, 1655).

In the United Kingdom today, with much better record-keeping and a permanently established army, proving one's service is much more straightforward than it was during or after the Civil Wars. The government, through consultation, has taken a very liberal view as to what constitutes military service in determining whether you are a veteran or not. The qualification is just 1 day's military service. Yet claimants still need to have the medical evidence of the injury occurring and the extent of the award is based on an assessment of that injury's severity. Therefore, in some ways, the process today remains similar to the process of submitting petitions. Nowadays, claimants are not so subject to the judgment of their local communities, but the methods of compensation do discriminate. They are separated between those that are attributable to military service, which are covered by the Armed Forces Compensation Scheme, and wider ill-health, which is essentially covered by the Armed Forces Pension Scheme as an occupational pension. So, as an example, the Armed Forces Compensation Scheme excludes injuries occurring outside of work, including travel to and from work. It also excludes conditions that are attributable to the consumption of alcohol or tobacco, or those that are self-inflicted (AFCS, 2005). But personnel are entitled, under their pension scheme, to receive benefits if those can be attributed to ill-health. Therefore, support is no longer subject to such social judgment as in the seventeenth century, but there are still clauses that determine whether one's condition is caused by military service or not.

There is evidence in some petitions that mental as well as physical injuries could meet the conditions for a soldier to make a successful claim. There is a general consensus amongst medical historians that it is anachronistic to apply present-day diagnoses to health conditions in the past. Such retrospective diagnoses inherently fail to take the experiences, modes of expression and contexts of those living in the past seriously. Instead, it is argued, we should seek to understand the names and descriptions given to ailments by contemporaries on their own terms. Furthermore, retrospective diagnoses contain the in-built assumption that present-day medical knowledge is static and uncontested and somehow detached from current contexts and concerns (Foxhall, 2014, pp. 356–358). The discourse surrounding retrospective analyses has been especially visible, even heated, in considerations of psychological conditions throughout history, including those resulting from warfare. Few now dispute that the circumstances and environment of combat cause complex and unique responses that manifest themselves in numerous symptoms, of which those associated with the condition most familiar to modern society—post-traumatic stress disorder (PTSD)—represent only some (Hyatt-Burkhart and Lopez Levers, 2012, pp. 23–24; Buck, 2012, p. 434). PTSD is not simply just the renaming or better understanding of an enduring affliction but a condition where social and cultural concerns are deeply embedded in its symptoms and diagnosis (Rees, 2022, p. 23). Indeed, the recent and growing interest in understanding historical war trauma is in itself reflective of current socio-cultural contexts. As Pamela Moss and Michael J. Prince have argued, “Such interest has no doubt been buoyed by the increase in number of armed conflicts worldwide, the rise of national identity-based separatist wars, the global circulation of detailed descriptions and images of war, and the media coverage of war crimes trials” (Moss and Prince, 2014, p. 7).

Nevertheless, there are petitions, with “tantalizing references to troubled emotions potentially indicative of such a condition (i.e., combat-related trauma)” (Pells, 2021, p. 130). The petition of John Cornelius listed his many physical injuries, but then added that he was “not only decripate, But hath alsoe lost the use of his reason, by his greife” (Cornelius, 1672). He was granted an immediate gratuity of £2 while the justices investigated awarding him a regular pension. William Summer petitioned that his house was ransacked and demolished during the royalist assault on Leicester, with his son killed defending the town. He claimed that his wife “with the fright whereof… hathe been distracted ever synce.” His petition was successful too (Summer, 1645–47). The husband of Goodwife Horne returned from the wars “a distracted man and in his madness did sett on fire and burne his owne house,” rendering them both homeless and prompting the justices to award Goodwife Horne a sizeable single sum payment of £6, 13 shillings and 4 pence (Horne, 1662). Care of those with mental injury usually fell upon their families, but the seventeenth-century justices' treatment of these individual cases might compare favorably with those veterans stigmatized for war-induced psychological trauma after the American Civil War who “were among the least likely to receive benefits,” or their counterparts during the First World War who were dismissed as “malingering” (Fuzal, 2024, pp. 132–133). The subject of mental injury in today's Armed Forces has been the subject of a great deal of debate, including themes such as causation, psychosocial context, treatment, recovery, resilience, and rehabilitation (Buck, 2012, pp. 434–453). For those who suffer from them, the mental health consequences of war can be just as important, or indeed worse than their physical injuries. Nowadays, we are able to recognize this more publicly, and there are mechanisms for support built into the Armed Forces Compensation Scheme which acknowledge the psychological as well as the physical consequences of war. Yet then as now, much depends on the societal construct of mental illness in the wider context.

We will never know how many or what proportion of applicants for seventeenth-century military welfare made successful claims because of the incomplete nature of document survival. It is probable that there is a bias among surviving documentation for the successful petitions to have been preferentially kept and preserved, while those that were unsuccessful were more likely to have been discarded. There are some surviving examples of unsuccessful petitions, where a court official appears to have written across the bottom, “Not successful,” or “Not granted.” Likewise, there are some successful petitions that have been endorsed with an amount at the end of the document, but this practice is inconsistent, so statistical analysis of these would be too problematic to yield meaningful results.

### Processes by which claimants accessed military pensions

Most seventeenth-century petitioners were either illiterate, or unskilled in drawing up a formal legal document. Therefore, they would enlist the help of a legally trained scribe, paying them to draft their petition in the correct manner, to meet the keywords and conditions in the legislation expected by the justices. This means that petitions do not represent the straightforward, unmediated testimony of the petitioner themselves. Rather, the authorship of petitions was more hybrid; a collaborative process, in which the scribes would advise on form of words and select from the oral testimonies of claimants. Then, claimants needed to travel to their county's court of quarter sessions where their petition was heard. If they had mobility issues, that could present problems and travel could be expensive. Then, they were in court, where they had to answer questions from the justices of the peace and to prove their claim. To help with this, claimants needed certificates signed either by their military commander or the medical practitioner who had treated them. For some petitioners, this was made problematic if these individuals were already dead. Widows also encountered serious difficulties proving that their husbands had been killed in service, especially if they had served abroad in Scotland or Ireland and had been absent for many years. “Missing in action” was not sufficient grounds to receive a pension. Attendance of the claimant in person, and the very pubic nature of the quarter sessions meant that if there was something untrue in the petition, there was a good chance of this being discovered and complained about (Bowen, 2024, pp. 51–54).

Then petitioners competed with other claimants for the limited sums available from the county's collected funds. Petitioners needed their case to stand out and be memorable to the justices. Even when petitioners were successful in obtaining an order for a pension, they still had to ensure that they actually received the money. There were many reasons why an order would not be carried out. Often the county treasurers encountered difficulty collecting the tax which funded the pension scheme. Parishes fell behind in making their payments. Poorer counties such as Lancashire, where the multiple harvest failures in the late 1640s were particularly harsh, failed to raise sufficient sums for their vast numbers of claimants. They directed worthy recipients onto parish poor relief instead. Petitioners needed to be patient and persistent. In order to succeed in collecting her pension, one war widow, Mary Burden, petitioned seven times between 1653 and 1659, three times doing so with a supporting letter from Oliver Cromwell himself (Burden, 1653–9). Maimed soldiers were sometimes required to strip and show their wounds in court to refute the consistent suspicions of justices and onlookers that they were somehow counterfeiting their injuries. Justices often looked for reasons to declare pensioners fit for work in order to conserve funds. Some soldiers might have relished the chance to display their wounds as badges of honor in service to a cause, but some likely found the requirement of stripping unwelcome and invasive. For example, Ellis Evans of Penmorfa had been “shott through his yard,” with bullets “in his privie members & other places of the body very dangerous” (Evans, 1660; Bowen, 2022). Nowadays, the medical assessment process remains similar in some respects, but is certainly much less public and intrusive. Those discharged from military service on medical grounds have a medical board where military doctors make a record of their medical condition. This is part of the process of determining the tariff. There is a mechanism for appeal if the claimant feels that the assessment has not been fairly graded.

The process of claiming by those who were no longer able to cope, or who faced a fall in social status, may have incurred stigma or shame at their poverty or their disability. For this reason, many petitioners were careful to point out that they had maintained themselves and their families for as long as they had been able to do, before resorting to petitioning for help. Christopher Ambler related how despite being many times wounded in royalist service, he had maintained his family of 11 children by working as a miner until he was over 70 years old, when a debilitating accident in the coal pit finally prevented him from laboring (Ambler, 1685).

Justices of the peace tended to be well-informed about how to treat those who petitioned them on the basis of disability. There were manuals and books of instructions written for justices giving advice on this, suggesting that disabilities were recognized. One especially moving example is the petition of Elizabeth Bradley, the widow of an infirm Yorkshire royalist captain. Elizabeth regretted that she was no longer physically able to look after her husband. She had likely been suffering from breast cancer and had endured a mastectomy, but did not want her neighbors to know about this. Her petition twice requested the justices to keep her claim confidential. As her family already faced the prospect of slipping from their gentry status, this suggests a degree of shame, hesitancy, and upset (Bradley, 1681).

This theme remains an ongoing consideration for claimants today. One of the challenges in the military community is because fitness and health are central components of being able to fulfill one's duty, there is a psychological consequence of not being fit and either needing time to recover, or, leaving the service as a consequence of an injury or ill health. The question of stigma, and the social consequences of seeking help, can also be experienced by those suffering mental health consequences from their wartime service. The United Kingdom's Armed Forces are striving to reduce the stigma associated with mental ill-health in order to encourage those people who need support to request it (Randles and Finnegan, 2022, pp. 99–104).

In addition to applying for county pensions, Civil War veterans sought to access more informal forms of relief by appealing to the charitable and patriarchal instincts of landowners. Royalist veterans might appear before the gates of the royalist gentry on an auspicious day in the calendar such as Christmas, the celebration of which gratifyingly irritated the parliamentarian authorities (Moore, 1686). Some royalist officers such as James Compton, Earl of Northampton and Captain Bartholomew Gidley signed numerous certificates for their former soldiers (Stoyle, 2021, pp. 81–100). We know that wealthy aristocrats were approached for charitable support. Margaret Cavendish, duchess of Newcastle reported one such encounter in her history of the life of her husband, William, who had been the royalist general in northern England. She recalled how after the Restoration her husband turned away one royalist widow who importuned him in person:

“A Soldiers Wife, whose Husband had been slain in my Lord's Army, came one time to beg some relief of my Lord; who told her, That he was not able to relieve all that had been loyal to his Majesty; for said he, my losses are so many, that if I should give away the remainder of my Estate, my Wife and Children would have nothing to live on: She answer'd, That his Majesty's Enemies were prefer'd to great Honors, and had much Wealth: Then it is a sign (replied my Lord) that your husband and I were Honest Men.”

(Cavendish, 1675, p. 242).

On the parliamentarian side, Oliver Cromwell wrote in support of many claimants, most conspicuously in the 2 years prior to his dissolution of the Rump Parliament in 1653 (Pells, 2023). His predecessor as commander-in-chief of Parliament's New Model Army, Sir Thomas Fairfax, granted a rent-free farm to John and Elizabeth Denonley in his will. Fairfax noted this was in recognition for John having “received a maim in my service disabling him to earn his living” (Markham, 1870, p. 445).

In comparison, today's veterans benefit from a wider range of national charities, including expert supporters to assist them in navigating complicated government bureaucracy. Government and charities are better organized to support the needs of service personnel and their families. One prominent example of a rich aristocrat supporting Armed Forces personnel is the late Gerald Cavendish Grosvenor (1951–2016), Duke of Westminster, who from 2011 donated a substantial amount of money to enable the Defense and National Rehabilitation Center at Stanford Hall to be built, which was opened in 2018 and has revolutionized the opportunity for the rehabilitation of injured service personnel. Furthermore, the charity, Help for Heroes, was created in the peak of the intensity of the battles in Iraq and Afghanistan, and has raised large sums for service personnel and their families, in addition to the existing Armed Forces charities. Individual regimental charities often created bespoke funds for those injured in Afghanistan or Iraq. This raises the question, what is the limit of government responsibility, and what should be the remit of charities to fill any gaps? In the United Kingdom there is a very established role for charities. This has long enabled the United Kingdom to offer lower pensions than comparable democracies such as the United States of America and Australia, but these charities can often also hold those in authority to account to make sure that the government meets its obligations (Crotty et al., 2021, p. 33).

### Motives of the state in supporting or denying claimants

The question of whether to grant or withhold state welfare to veterans and their families has always been a political decision. A spectrum of authorities' motives range from upholding moral righteousness, through to concerns about maintaining order in society, to anxieties for self-preservation. The day after the Battle of Edgehill, the House of Commons, uncertain of its own future, decided to extend eligibility for pensions to include not just Parliament's wounded soldiers but also to include the widows and orphans of those slain in their cause (Commons' Journals, 1642). This was unprecedented and its implications were likely not fully appreciated at the time. As was to be the case subsequently with the American Civil War, the leaders on both sides badly underestimated the war's length and size. The Long Parliament in the 1640s and the Union in the 1860s both hoped that the promise of pensions would encourage men to volunteer, sure in the knowledge that their families would be looked after if they were killed or incapacitated (Fuzal, 2024, pp. 16, 42). Colonel Algernon Sidney made this point when supporting the petition for a pension of Elizabeth Newson, the widow of one of the gunners at Dover Castle, writing that the “concession hereof I doubt not will appeare to be not only an acte of Justice & mercy but an encouragement to others to adventure their lives for the State in hope of the like releife for theirs” (Newson, 1650).

Owing to the promises made by Parliament to mobilize popular support, there was also a widespread contemporary sense of rights and entitlement; that relief was a just reward for those who had endured loss and sacrifice for “the cause.” Anne Aliston petitioned that her husband Roger had been “willing to venter his life in the Cause of Christ,” but that it “hath pleased God that in the service he hath lost his life.” This had left Anne unable to support her “three very small fatherlesse infants.” Anne boldly requested her husband's late arrears of pay “which in right was due to him at his death.” Members of the county committee of Suffolk endorsed Anne's petition ordering her an immediate payment of 20 shillings, adding “this petitioner's case is lamentable” (Aliston, n.d.). This is suggestive of the beginning of an understanding of the reciprocal obligation between the state and those risking their lives in its service.

Some petitioners therefore took pride in making their applications. Some stressed that they had volunteered, or served constantly from the very beginning of the wars. Trooper Rowland Harrison described his service at great length, narrating his participation in the war's most famous battles and his many daring escapes from captivity. There is a sense that Harrison enjoyed recounting his story and that it may have grown in the telling. The local gentry who endorsed his claims signed that “they had heard the same confirmed for more than 30 years” (Harrison, 1685). Pensions vindicated the military service and honor of their recipients, and were a marker of support for loyalty that upheld individuals' local status and reputation. There is some evidence that veterans gathered on anniversaries to take pride in their service and commemorate their fallen comrades. The parish of St. Botolph without Aldgate in London held an anniversary sermon to celebrate the parliamentarian victory for many years after the First Battle of Newbury, at which veterans of the local red regiment of the London trained bands attended (Peck, 2021, pp. 142–144). Nowadays, Remembrance Day provides an opportunity for the nation to recognize the value of military service. Veterans take pride in attending memorial services and choose to wear their medals, while organizations such as the British Legion promote veteran sociability. With the repatriation of British service personnel killed overseas since the Falklands War, their bodies are no longer the property of the state as established by the Imperial War Graves Commission in 1917. Consequently, there has been a move toward remembrance becoming more shaped by their families and descendants. As Ian Atherton points out: “Commemoration and memorials, having been nationalized in the early twentieth century, have been personalized in the twenty-first” (Atherton, 2024, p. 305).

Another reason for granting military welfare was that the social elite, as represented by Members of Parliament and justices of the peace were very concerned to maintain the social order. After the first major engagement at the Battle of Edgehill, there was a dawning realization about the numbers of the maimed and bereaved that were going to need support. A sudden rise in numbers of people in poverty had the potential to cause social unrest, especially during the harvest failures of the late 1640s, so granting pensions and military welfare was one means of mitigating this dangerous impact of war. There was also an element of propaganda in the paper war fought between the newsbooks and pamphlets on both sides. Royalists and parliamentarians alike wanted to be seen to support their maimed soldiers and war widows, although the royalists did not legislate for any nationwide support for their war widows during the war itself. Each side accused the other of failing to care for those wounded and bereaved in their cause. Some claimants, such as the parliamentarian Captain Humphrey Tudman, understood this well enough to attempt to shame the authorities into action. Tudman petitioned one of Parliament's committees in 1653 that he could no longer support his orphaned nephews, who were the sons of the famous parliamentarian, Colonel John Fox. Unless Parliament settled “their great wants,” Tudman would be forced to release them onto the streets to beg for their living, which would enable “the Comon wealthes enemies [to] say in reproach and especially in ye County where his service was soe eminent Theis are ye Children of Colonell ffox” (Tudman, 1653). He invited them to consider how this would reflect on Parliament's honor.

The changing governing regimes of the mid seventeenth century dictated who should receive welfare, politicizing the pension scheme. Both parliamentarians and royalists were only prepared to award support to those who had fought on their own side. With the return of Charles II at the Restoration in 1660, a vast new wave of royalist claimants, long denied pensions, now jostled to displace the parliamentarian pensioners. The speed with which this occurred depended very much on the county administration. In some places, such as Kent, this transition was very sudden, perhaps owing to the large numbers of royalist justices of the peace returning to the quarter sessions bench with scores to settle. In 1662 in the West Riding of Yorkshire, two returning royalist justices announced that they would imprison any former parliamentarian soldiers found to be still in receipt of a pension. And that such individuals would remain in prison until they had repaid the full sum to the county treasurer. In other counties such as Essex, which had more of a mixture of justices, with some former parliamentarians persisting among them, this transformation was slower and more gradual, taking several years before all of the parliamentarians had been removed. Some claimants might hope that with the passage of time people had forgotten for which side they had fought. Others among the parliamentarian soldiers who had fought in Ireland were permitted to retain their pension, as they might claim by fighting the Irish rebels they had not been in arms against the English crown. There were also some parliamentarians who managed to retain their pensions by claiming that they had served under General George Monck in 1660, the military commander who did most to instigate the Restoration of Charles II. Nathaniel Lingard is one such example, who received a pension of 40 shillings in 1671 for having served under Monck's command, thereby claiming to be “instrumental in the happy restoration of his Majesty” (Lingard, 1671).

There are two comparative perspectives on why the United Kingdom grants veteran welfare today. One is in order to guarantee that the Armed Forces are motivated to fight, and have the support of their families to do so. During wartime, there is additional pressure to ensure that those people who are inevitably going to lose from the consequence of war are properly respected and cared for, so that the next group of soldiers, sailors or airmen will step up to join the fight. That is the short-term cost. But the real question is the motive behind the long-term cost, because actually pensions and other legacy costs extend out over a long period of time. This question enters the realm of politics rather than military capability, and what it is that motivates politicians to keep the Armed Forces and veterans on their side. Examining the benefits provided to veterans across different nations and how they have played out in politics since World War Two, maintaining the opinion of the veteran community has become a much more significant political imperative than it was to seventeenth-century politicians (Brooke-Holland, 2024).

During and after the Civil Wars there was considerable variation in the level of sums awarded to soldiers and widows. Maimed officers and the widows of officers were usually of higher social standing than the rank-and-file soldiers. As a result, they tended to receive higher pensions. An average annual pension for a soldier was usually around £2. This only amounted to about a fifth of what was needed to maintain a poor man's family for a year, but it was only ever intended as a supplement to what might be obtained by other means, including through paid employment, parish poor relief, neighborly assistance and customary rights. Yet the annual pensions of officers or officers' widows could be as high as £5 or even £10. The reason for the pension being more linked to social status than severity of need was a recognition that it was more expensive for those higher in the social hierarchy to maintain their position. Those petitioners who were landed gentlemen were also more likely to be well-connected and to know the justices of the peace personally, perhaps improving their chances of making a successful claim.

One of our most extreme examples is Grace, the widow of the royalist Colonel Robert Portington. Robert endured 12 years of imprisonment for his ardent royalism in Kingston-upon-Hull. Upon his release in 1660, he died of gangrene resulting from a bite from his pet monkey while on board a ferry boat crossing the River Ouse. Although Robert had not died as a result of his military service, Charles II was anxious to limit the fall in social status brought about by Grace's widowhood. He ordered that the West Riding of Yorkshire's ratepayers furnish her with an annual pension of £20. She collected this every year between 1665 and her death in 1680, amounting to £300 in total. This colossal sum dwarfed the grand total of £4, 11 shillings and 4 pence awarded to all other royalist widows in the West Riding combined (Portington, 1665–80). Nowadays the tariff of a pension is based on assessments of function and loss, but the actual calculation is also based on the individual's salary. As military rank affects one's salary it might still be argued that today's levels of pension still reflect social status, although current pensions are shorn of the political considerations so evident during and after the Civil War period.

## Concluding remarks

The Civil War Petitions project has established that military welfare and medical care has had a much longer history in the United Kingdom than many people would assume. The sociological theory that twentieth-century advances in military welfare were driven through powerful collective action by and on behalf of veterans can now be seen to possess antecedents 3 centuries earlier, with the New Model Army playing a major part in shaping the Interregnum's political regimes (Crotty et al., 2021). The argument that the provision of welfare to veterans' families declined during the 2 centuries that followed the Restoration challenges the notion that war is uniformly “associated with the most socialized and most valued members of a society” (Centeno and Yang, 2020, p. 319). It also highlights how the emergence of the state and the social contract which bound state and citizens together was a far from unilinear process. Instead, another recent work takes a global approach to twentieth-century veteran welfare, combining the methods of history and political sociology to call for more comparative veteran studies. Drawing upon collective action theory, it concludes that those veterans who succeeded in improving their lot did so largely by forcing their agendas through lobbying, protesting, and mobilizing civilian support. Victory in war did not always guarantee generous treatment from the state, while poor treatment did not always follow on from defeat. Rather, veterans' successes were the result of veterans becoming more politically powerful by organizing public support to maximize whatever opportunities were available to them within their state's political structure (Crotty et al., 2021, p. 161).

The stories uncovered on the project's website are very human and quite relatable for modern audiences. By sharing and publicizing them, the sacrifices of maimed soldiers and war widows of this foundational series of conflicts might be acknowledged and remembered, so that their sacrifices were not all for nothing. We should remember that British military welfare did not begin with Florence Nightingale or with the reintroduction of pensions for war widows at the end of the South African War. This discussion about the resonance of the Civil War experience for today might act as a bridge between the past and the present, to broaden awareness among schools, veterans, military families and the Armed Forces community of what we might learn from the suffering and sacrifices endured by soldiers, widows, and orphans of the seventeenth century.

With this in mind, we might reflect on recent advancements in mobilizing political and public opinion toward collective action to improve the style and types of services offered to today's Armed Forces and veterans who have made a very significant sacrifice on behalf of the state. These improvements are not only in regard to access to health services but much more to do with wider welfare support. The National Health Service is now commissioning particular routes of healthcare that reflect military service, both in mental and physical health, recognizing that there are unique needs for Armed Forces personnel and veterans in accessing healthcare (NHS Support, 2024). This is also reflected in the interface between healthcare and social care, which is often what charities help deliver when individuals fall between that gap.

Lastly, we might also reflect on how the care and welfare offered to British forces and veterans compares with that offered to others in Western democracies such as France, Germany, Australia, Canada, and the United States of America. The failure of UK governments to provide as generous veteran welfare as in some other western democracies certainly seems indicative of a significant breakdown of the social contract between the state and those who fight in its service. However, the situation is more complex than that. The charities that have stepped up to fill the void left by inadequate state veteran welfare have taken on the language of the social contract. For example, the charity “Help for Heroes” emphasizes that the responsibility to repay veterans for their service lies with society at large: “sometimes, what you give to your country comes at a huge cost… Having sacrificed so much on our behalf, we must not leave them to fight alone. We all have a duty to stand with those who served and make sure they and their families get the life-changing support they urgently need” (Help for Heroes, 2025).

The fact that military charities are successful suggests that society has embraced this language and taken it to heart. In a way, this is a success story for the social contract. Widening the responsibility for veteran welfare from government to wider society is no doubt helpful for governments. It tends toward mitigating the responsibility of governments toward their veterans. This suggests the UK is returning to a pre-1593 situation, when military welfare was the responsibility of charity, such as that bestowed by the Church, bequests, almshouses or private institutions such as the Lord Leycester's Hospital, established in Warwick in 1571 and still operational today. Early modern charities often expressed religious and moral motivations, whereas today's charities use more secular language, focused on the responsibility of the donor to perform a duty of care for those who have risked their lives for them. If the social contract has failed in its formal sense, with charities stepping up to compensate for the state's inadequacy, the language and spirit of the social contract has persisted. This suggests that once instituted, the idea of a social contract is difficult to roll back entirely. The alternative of ignoring the wounded and injured dishonors their service and endangers the state they serve (Fuzal, 2024, p. 171). Because as Peter Reese, of the Royal Army Education Corps, wrote: “Any state which short-changes its guardians deserves neither liberty nor safety” (Reese, 1992, p. 273).

## Data Availability

The datasets presented in this study can be found in online repositories. The names of the repository/repositories and accession number(s) can be found below: https://www.civilwarpetitions.ac.uk/.
